# Gut–Liver Axis as a Therapeutic Target for Drug-Induced Liver Injury

**DOI:** 10.3390/cimb46020078

**Published:** 2024-02-01

**Authors:** Wenjing Tao, Qiwen Fan, Jintao Wei

**Affiliations:** Hubei Key Laboratory of Animal Embryo and Molecular Breeding, Institute of Animal Husbandry and Veterinary, Hubei Academy of Agricultural Sciences, Wuhan 430064, China; taowenjing1127@hbaas.com (W.T.); qwfan11@hbaas.com (Q.F.)

**Keywords:** drug-induced liver injury, gut–liver axis, gut microbiota, metabolites, bile acid

## Abstract

Drug-induced liver injury (DILI) is a liver disease that remains difficult to predict and diagnose, and the underlying mechanisms are yet to be fully clarified. The gut–liver axis refers to the reciprocal interactions between the gut and the liver, and its homeostasis plays a prominent role in maintaining liver health. It has been recently reported that patients and animals with DILI have a disrupted gut–liver axis, involving altered gut microbiota composition, increased intestinal permeability and lipopolysaccharide translocation, decreased short-chain fatty acids production, and impaired bile acid metabolism homeostasis. The present review will summarize the evidence from both clinical and preclinical studies about the role of the gut–liver axis in the pathogenesis of DILI. Moreover, we will focus attention on the potential therapeutic strategies for DILI based on improving gut–liver axis function, including herbs and phytochemicals, probiotics, fecal microbial transplantation, postbiotics, bile acids, and Farnesoid X receptor agonists.

## 1. Introduction

Drug-induced liver injury (DILI) refers to the liver damage induced by a variety of commonly used prescription and nonprescription medications, herbs, and dietary supplements, as well as illegal drugs and novel agents [1,2]. Epidemiological studies have shown that the annual incidence of DILI varies from 2.3 to 23.8 per 100,000 individuals in different countries, but it is accepted that the actual DILI incidence is likely higher than that reported [3,4]. The most common clinical biomarkers of DILI are elevations in serum levels of alanine aminotransferase (ALT), aspartate aminotransferase (AST), and alkaline phosphatase (ALP), usually in association with increased serum total bilirubin (TBIL) levels [5]. However, because there is a lack of special biomarkers for DILI, the diagnosis of DILI is difficult and mainly depends on a high degree of suspicion and close follow-up, as well as careful exclusion of all alternative causes of liver injury [6]. Most patients with DILI can recover quickly after stopping the use of causative drugs, whereas some may develop chronic DILI, which refers to DILI that lasts for more than one year [7]. About 10% of DILI patients with concomitant jaundice may go on to need liver transplantation or even die [6]. A two-year follow-up investigation showed that DILI directly or indirectly contributed to fatality in 7.6% of patients [8]. Moreover, as one of the most common and severe clinical adverse drug reactions, DILI is a major cause of drug withdrawal from the market, leading to failures in the development of new drugs and economic loss for the pharmaceutical industry [9]. According to the pathogenesis, DILI is classified into direct, idiosyncratic, and the newly proposed indirect liver injury [10]. Direct DILI is induced by agents or their metabolites that are intrinsically toxic to the liver in a dose-dependent manner, and it usually, predictably, happens within a few hours of exposure [10]. For example, high dosages of acetaminophen (APAP), amiodarone, aspirin, or various anti-cancers drugs have direct hepatotoxicity [10]. In contrast, idiosyncratic DILI only occurs in rare patients with a variable latency period from a few days to several weeks, and its onset is unpredictable because it is driven by the interplay of multiple factors, including drug properties, host susceptibility, and environmental conditions [11]. Several antibiotics like amoxicillin-clavulanate, cephalosporins, fluoroquinolones, and macrolides may induce idiosyncratic liver toxicity [12]. Indirect DILI is associated with the action of the drugs rather than their intrinsic hepatotoxicity or idiosyncratic effect [13]. For example, immune checkpoint inhibitors used for cancer can lead to hepatitis and hepatocyte death by activating the T-cell response against malignant cells [14]. To date, the exact pathogenesis of DILI remains largely unknown, so it is difficult to predict the occurrence of DILI or develop effective preventive and therapeutic interventions.

The gut–liver axis refers to the reciprocal interactions between the gut and the liver [15]. Under healthy conditions, dietary constituents, gut-derived bioactive substances, and pathogenic or toxic compounds are delivered to the liver to be metabolized or detoxified through the portal vein [16]. In turn, the bile acids (BAs) and antimicrobial peptides generated in the liver are transported to the gut through the bile ducts, ultimately exerting an influence on gut microbiota, as well as intestinal epithelial cells and immune cells [17,18]. Growing evidence indicates that a disturbed gut–liver axis is involved in the onset and development of various liver diseases, such as alcoholic liver disease (ALD), non-alcoholic fatty liver disease (NAFLD), primary sclerosing cholangitis (PSC), and primary biliary cholangitis (PBC) [15,19]. Recently, an accumulating number of studies have demonstrated that the development of DILI is often accompanied by gut–liver axis dysfunction, and studying the bidirectional relationship between the gut and the liver is of great significance for the treatment of DILI [20,21]. The role of the gut–liver axis in liver diseases has been summarized in several recent reviews, but DILI is often not included in the liver diseases discussed, or confused with non-drug-induced acute liver injury. In this review, we will focus on the results from both preclinical and clinical studies on the role of the gut–liver axis in the pathogenesis of DILI. Moreover, we will summarize the potential therapeutic strategies for DILI on the base of improving the function of the gut–liver axis.

## 2. The Gut–Liver Axis in DILI

**Figure 1:**
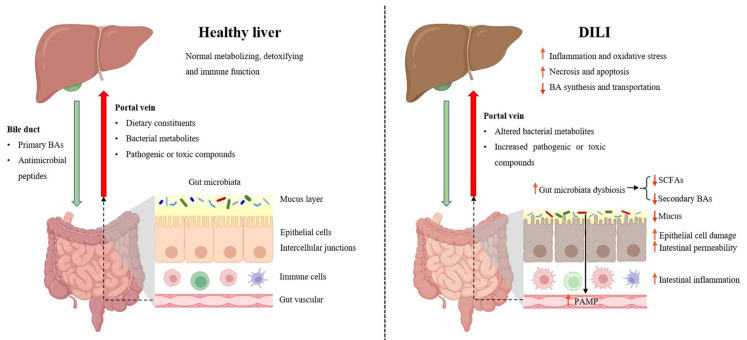
The gut–liver axis in DILI. BA, bile acid; DILI, drug-induced liver injury; PAMPs, pathogen-associated molecular patterns; SCFAs, short-chain fatty acids (Created using BioRender.com (accessed on 28 November 2023)).

The term “gut–liver axis” highlights the anatomical and functional crosstalk between the gut and the liver, and maintaining the hemostasis of the gut–liver axis is critical for host health [15]. The elements of the gut–liver axis consist of gut microbiota, intestinal barrier function, bacterial products and metabolites, and BAs, and their alterations are critically involved in the progression of DILI (Figure 1).

### 2.1. Gut Microbiota

There are more than 100 trillion microorganisms residing in the human intestine, including bacteria, fungi, viruses, and archaea, and the total number of genes in the intestinal flora is 100 times greater than that in the human genome [22]. The gut microbiota plays an important role in maintaining host health by extracting energy and nutrients from food, metabolizing drugs and xenobiotics, regulating immune responses, and preventing pathogen colonization [23]. The gut microbiota composition is dynamic, and the disruption of gut microbiota homeostasis is involved in the initiation and development of a variety of diseases [24].

Recent studies have revealed a significant role of the gut microbiota in the pathogenesis of DILI [20,21]. Clinical studies reported that patients with DILI induced by various drugs had decreased richness and diversity in the gut microbiota compared to healthy people [25,26]. At the phylum level, the abundance of Firmicutes was lowered, whereas the abundances of Bacteroidetes, Proteobacteria, and Actinobacteria were increased in the feces of DILI patients. At the genus level, DILI decreased the relative abundance of *Acetobacteroides*, *Bacteroides*, *Bifidobacterium*, *Blautia*, *Caloramator*, *Coprococcus*, *Flavobacterium*, *Lachnospira*, *Natronincola*, *Oscillospira*, *Pseudobutyrivibrio*, *Shuttleworthia*, *Themicanus*, and *Turicibacter*. The clinical trials conducted by Sun et al. found that antithyroid drugs could alter the gut microbiota composition, and a Spearman’s correlation analysis showed that the degree of liver injury was positively correlated with the abundances of *Blautia*, *Dorea*, and *Streptococcus*, and was negatively correlated with the abundances of *Faecalibacterium* and *Bacteroides* [27]. Consistent with these results, Sun et al. found that the gut microbiota constituents showed a change in DILI rats exposed to antithyroid drugs compared with the control group, and the changed abundances of several genera of gut microbiota were correlated with the liver injury induced by the antithyroid drugs [27]. It has been found that the richness and diversity of gut microbiota was increased in APAP-treated mice, and the gut microbiota composition of APAP-treated mice was distinctly separate from that of the control mice [28,29]. Specifically, at the phylum level, the abundances of Cyanobacteria, Deferribacterota, and Desulfobacterota were increased, and the abundance of Firmicutes was decreased by APAP treatment. At the genus level, the abundances of *Bacteroides*, *Blautia*, *Colidextribacter*, *Enterococcus*, *Erysipelatoclostridium*, *Eubacterium_brachy_group*, *Eubacterium_fissicatena_group*, *Eubacterium_nodatum_group*, *Family_XIII_AD3011_group*, *Gordonibacter*, *Mucispirillum*, *norank_f_Eubacterium_coprostanoligenes_group*, *norank_f_norank_o_Clostridia_UCG-014*, and *Oscillibacter* were increased, and the abundances of *Bifidobacterium*, *Candidatus_Saccharimonas*, *Dubosiella*, *Lactobacillus*, *Odoribacter*, and *Prevotellaceae_UCG-001* were decreased by APAP treatment. Additionally, ampicillin aggravated APAP-induced liver injury by reducing the diversity and altering the composition of the gut microbiota [30]. It has also been found that other drugs, such as methotrexate (anti-cancer drug), tacrine (anti-Alzheimer’s disease drug), and triclosan (antimicrobial ingredient) could change the gut microbiota composition of mice or rats, and the alterations in gut microbiota are closely correlated with the liver injury induced by these drugs [31,32,33]. The summary of the alterations in gut microbiota in DILI is presented in Table 1.

The gut microbiota could influence the hepatotoxicity of several drugs, thereby affecting the development of DILI. Schneider et al. analyzed data from 500,000 UK Biobank participants and found that participants with intestinal microbial dysbiosis, which is induced by long-term intake of proton pump inhibitors or antibiotics, have an increased risk of developing acute liver failure [34]. Schneider et al. used male Nlrp6^−/−^ mice as an intestinal dysbiosis mouse model and found that the degree of APAP-induced liver injury was higher in the Nlrp6^−/−^ mice than in the wild-type mice, whereas fecal microbiota transfer (FMT) led to increased severity of APAP-induced acute liver failure transmitting from Nlrp6^−/−^ mice to wild-type mice [34]. These results suggested that intestinal microbial dysbiosis could increase the risk of DILI. Interestingly, the hepatotoxicity of some drugs may change due to the diurnal concussion of the gut microbiota. The mice treated with APAP at zeitgeber time 12 (ZT12) (8:00 p.m.) showed more severe liver injury compared with that at ZT0 (8:00 a.m.) [35,36]. However, after antibiotics treatment, the enhanced liver injury in the mice treated with APAP at ZT12 was abolished. Moreover, the antibiotic-treatment mice that received ZT12 cecal content showed a higher degree of APAP-induced liver injury than those that received ZT0 cecal content [36]. A 16S rRNA sequence analysis showed that the ratio of Firmicutes/Bacteroidetes was decreased in the cecal content of mice at ZT12 compared to that at ZT0 [36]. Specifically, at the phyla level, the abundance of Bacteroidetes was increased and the abundance of Actinobacteria was decreased in the cecal content of mice at ZT12 compared to that at ZT0. At the genus level, the abundances of *Alistipes*, *Bacteroides*, *Barnesiella*, *Pseudoflavonifractor*, and *Rikenella* were increased, and the abundances of *Lactobacillus* and *Enterorhabdus* were decreased in the cecal content of mice at ZT12 compared to that at ZT0. 

Taken together, these studies show that some drugs could change the gut microbiota composition of patients or animals, and in turn, the gut microbiota could affect the hepatotoxicity of these drugs and the severity of DILI.

### 2.2. Intestinal Barrier Function

The intestinal barrier can protect the body against the invasion of antigens, toxins, pathogenic bacteria, and microbial metabolites [37]. Intestinal permeability is determined by the presence of enterocytes and intercellular junctions, and the tight junction, consisting of several protein families, such as zonula occludens (ZO), occludin, and claudin, is a major junction between adjacent epithelial cells [38]. There is a mucus layer on the surface of the tight epithelium, and the mucus, produced by goblet cells, can promote the transport of luminal contents and enable the selective passage of substances [39]. Mucin-2 (MUC2) is a core component of mucus and the best-studied mucus protein, and its dysregulated production is related to various intestinal diseases [40]. A series of immune cells present in the intestinal epithelial layer and lamina propria maintain intestinal immune homeostasis. Impaired intestinal barrier function leads to the increase in inflammation in the intestine, as well as the translocation of microbiota, bacterial products, and metabolites to the systemic circulation and liver tissue, thereby contributing to systemic inflammation and the progression of various liver diseases [22,41]. Albumin is the major protein in human blood, and it can pass from the blood vessels into the gut lumen once the intestinal barrier is damaged, so fecal albumin level can be used as an indicator for the evaluation of intestinal permeability [42]. 

Sun et al. found that the intestinal barrier’s physical structure was destroyed, and the serum levels of FITC-dextran were increased in rats with DILI induced by antithyroid drugs [27]. Xia et al. reported that APAP significantly downregulated the mRNA expression of *occludin* and *MUC2*, and downregulated the protein level of claudin in mice [28]. Triclosan-treated mice showed damaged colon tissues, reduced colon length, and downregulated protein expression of ZO-1, occludin, and claudin 4, and downregulated mRNA expression of *ZO-1* and *occludin* in the colon, as well as increased fecal content of albumin [33]. Methotrexate-treated mice presented epithelial damage, goblet cell depletion, intestinal inflammation, elevated serum FITC-dextran level, and decreased protein expression of the intercellular junction, including ZO-1, Claudin-1, and E-cadherin [43]. These findings suggest that DILI is associated with a damaged physical, chemical, and immunological intestinal barrier.

### 2.3. Bacterial Products and Metabolites

In various liver diseases, including DILI, gut microbiota dysbiosis and intestinal barrier dysfunction contribute to the altered influx of pathogen-associated molecular patterns (PAMPs) and gut-microbiota-derived metabolites to the liver through the portal vein.

Lipopolysaccharide (LPS), a kind of PAMP known as bacterial endotoxin, is a structural component of the cell wall of Gram-negative bacteria and can be released into the circulation due to increased gut permeability (leaky gut) [44]. LPS translocated into the liver can bind to LPS-binding protein (LBP), the LPS–LBP complexes are recognized by the receptor CD14, and then LPS is presented to Toll-like receptor 4 (TLR4), leading to the activation of the myeloid differentiation factor 88 (MyD88) and nuclear factor-kappa B (NF-κB) signaling pathways, ultimately aggravating hepatic inflammation via promoting the release of proinflammatory cytokines, such as tumor necrosis factor-α (TNF-α), interleukin (IL)-6, and IL-1β [45].

A clinical study has shown that the levels of LPS in the plasma of patients with DILI induced by various drugs were eight-fold higher than those in healthy people, and the plasma levels of LBP and CD14 also significantly increased, thereby activating the hepatic macrophage in DILI patients [46]. The clinical trials conducted by Sun et al. found that antithyroid drugs could increase LPS levels in the feces and serum of patients, as well as activating the related four metabolic pathways, including LPS biosynthesis, LPS biosynthesis proteins, bacterial toxins, and bacterial invasion of epithelial cells [27]. 

Sun et al. also found that the LPS levels in feces and serum were increased in rats with DILI induced by antithyroid drugs [27]. Xia et al. found that serum LPS levels were elevated in DILI mice exposed to APAP, resulting in the upregulation of the hepatic mRNA expression of *TLR4* and *MyD88* [28]. Triclosan treatment for four weeks increased the LPS levels in the serum and feces of mice, which was likely due to a drastic increase in the abundance of Enterobacteriaceae (a family belongs to Proteobacteria, the major source of gut-derived LPS), and the increased LPS was translocated to the liver, thereby activating the LPS/TLR4 pathway to promote hepatic inflammation [33]. Methotrexate administration resulted in increased bacteria translocation from the intestine to the liver, as well as elevating serum LPS levels, thereby increasing the degree of inflammatory cell infiltration, and inflammatory cytokine expression in the liver of mice [43]. Luo et al. reported that plasma LPS levels increased in DILI rats exposed to genipin (a metabolite of geniposide, which is one of the major bioactive components of the traditional Chinese medicine *Gardeniae Fructus*) [47].

Short-chain fatty acids (SCFAs) are metabolic products generated by the gut microbiota through fermenting dietary non-digestible carbohydrates. Acetic acid, propionic acid, and butyric acid are the most abundant SCFAs presented in the intestine [48]. The major gut microbiota responsible for SCFA production include *Bacteroides*, *Bifidobacterium*, *Clostridium*, *Faecalibacterium*, and *Lactobacillus* [49]. SCFAs can provide additional energy for enterocytes and regulate the proliferation and differentiation of intestinal crypt stem cells, as well as exert anti-inflammatory effects in colonic macrophages and dendritic cells, contributing to the maintenance of intestinal homeostasis [50]. In addition, SCFAs can enter the circulation and exert an influence on organs beyond the gut, and the SCFAs transported into the liver can exert a therapeutic effect on various liver diseases [51].

Xia et al. found the concentrations of acetic acid, propionic acid, and butyric acid were decreased in the feces of DILI mice exposed to APAP, and these changes may be related to the depletion of intestinal SCFA-producing bacteria [28]. Li et al. reported that ampicillin treatment significantly aggravated APAP-induced liver injury, accompanied by decreased levels of butyrate, hexanoic acid, and valeric acid in the feces of mice, whereas butyrate supplementation restored serum butyrate levels, and improved hepatic necrosis and function via activating the nuclear factor E2-related factor 2 (Nrf2) signaling pathway, thereby protecting mice from ampicillin-aggravated APAP-induced liver injury [30]. Pirozzi et al. found that sodium butyrate normalized serum biochemical parameters related to hepatic function, attenuated the impairment of hepatic lipid metabolism, and reduced hepatic inflammation and fibrosis induced by valproate (an antiepileptic drug) in epileptic WAG/Rij rats [52]. In vitro studies also found that sodium butyrate decreased valproate-induced toxicity, lipid accumulation, oxidative stress, and mitochondrial dysfunction in human hepatoma cell line HepG2 and primary rat hepatocytes [52]. Luo et al. reported that *Gardeniae Fructus* caused liver injury and decreased the butyric acid production in the caecal contents of rats, whereas intragastrically administered butyrate ameliorated the hepatic inflammation and necrosis induced by genipin by promoting Nrf2 expression and reducing LPS translocation in rats [47]. It has also been demonstrated that butyrate protected HepG2 cells from genipin-induced cytotoxicity by enhancing Nrf2 expression [47].

Based on the above evidence, the development of DILI is related to the increased LPS translocation from the intestine to the liver, as well as decreased SCFA production.

### 2.4. BAs

BAs consist of primary BAs, synthesized from cholesterols in the hepatocytes, and secondary BAs, converted from primary BAs in the intestine [53]. The major primary BAs include cholic acid (CA) and chenodeoxycholic acid (CDCA) in humans, whereas CA and muricholic acid are the predominant primary BAs in mice [54]. There are two pathways for the biosynthesis of primary BAs, namely the classical pathway and the alternative pathway, initiated, respectively, by cholesterol 7α-hydroxylase and sterol 27-hydroxylase [55]. After being conjugated with glycine or taurine, primary BAs are stored in the gall bladder in the form of bile salts and enter the duodenum to participate in the digestion and absorption of lipids and lipid-soluble nutrients when a meal is consumed [56]. About 95% of BAs are reabsorbed before reaching the terminal ileum and transported to the liver to be recycled, whereas the remaining BAs enter the large intestine and are subsequently metabolized by the gut microbiota to secondary BAs [57]. Deoxycholic acid (DCA) and lithocholic acid (LCA) are the main secondary BAs in humans, whereas DCA, hyodeoxycholic acid, and murideoxycholic acid are the predominant secondary BAs in mice [54]. The main gut microbiota responsible for BA transformation include *Bacteroides*, *Bifidobacterium*, *Clostridium*, *Eubacterium*, *Escherichia*, and *Lactobacillus* [17,58]. The initial step of BA transformation is the hydrolysis and deconjugation of glycine or taurine-conjugated BAs catalyzed by bile salt hydrolases, then the deconjugated BAs convert to secondary BAs by undergoing additional microbiota-mediated reactions, such as 7α-dehydroxylation, dehydrogenation, and epimerization [59].

It has been demonstrated that BA metabolism and transport were disrupted in DILI patients, and impaired BA homeostasis was one of the mechanisms contributing to the progression of DILI [60,61]. Compared to healthy people, DILI patients had elevated serum levels of taurocholic acid (TCA), glycocholic acid (GCA), rochenodeoxycholic acid (TCDCA), glycochenodeoxycholic acid (GCDCA), taurodeoxycholate acid, glycodeoxycholic acid, taurohyocholate acid, tauroursodeoxycholic acid, and norcholic acid [25,62,63,64,65]. The serum levels of TCA, GCA, TCDCA, and GCDCA were positively correlated with the severity of DILI, and identified as potent markers for the diagnosis and severity discrimination of DILI [25,64]. Additionally, the ratio of primary BAs to secondary BAs in the serum of DILI patients was increased, and this change may be attributed to the reduced abundance of BA-transforming bacteria in the intestine of DILI patients [25]. 

Farnesoid X receptor (FXR), as the first described BA receptor, is mainly activated by the primary BAs, including CA and CDCA [66]. FXR is highly expressed in the liver and ileum, and normal FXR activity facilitates the regulation of BA metabolism and circulation, as well as the immune functions of the liver and intestine [66]. Yan et al. found that global Fxr-null (Fxr^−/−^) mice had more severe liver injury induced by APAP compared with wild-type mice, but hepatocyte-specific or macrophage-specific Fxr-null mice did not show increased sensitivity to APAP-induced hepatotoxicity, indicating that global FXR deficiency increased APAP-induced hepatotoxicity by disrupting the systematic homeostasis of BA [67]. Fibroblast growth factor 19 (FGF19) is produced and released by enterocytes located in the human terminal ileum in response to the activation of intestinal FXR [68]. The clinical studies conducted by Zhao et al. reported that patients with DILI induced by various drugs had increased serum FGF19 levels compared to healthy people, and FGF19 inhibited BA synthesis in the liver [25].

Total BA levels in the circulatory system were increased, and the serum levels of secondary BAs, including DCA and LCA, were decreased in mice with DILI induced by triclosan [33]. Takeda G protein-coupled receptor 5 (TGR5), as a BA receptor, is mainly activated by unconjugated secondary BAs, including LCA and DCA, and its activation plays a critical role in maintaining BA homeostasis and preventing hepatic inflammation [69]. It has been found that the hepatic mRNA expression of *TGR5* was downregulated in the mice with DILI induced by triclosan [33].

Collectively, these data demonstrate a close relationship between DILI and impairment of bile acid homeostasis, and drug-induce abnormalities in gut microbiota composition result in altered BAs levels and impaired BA-related signaling pathways.

## 3. Gut–Liver Axis-Based Therapeutic Approaches for DILI

**Figure 2:**
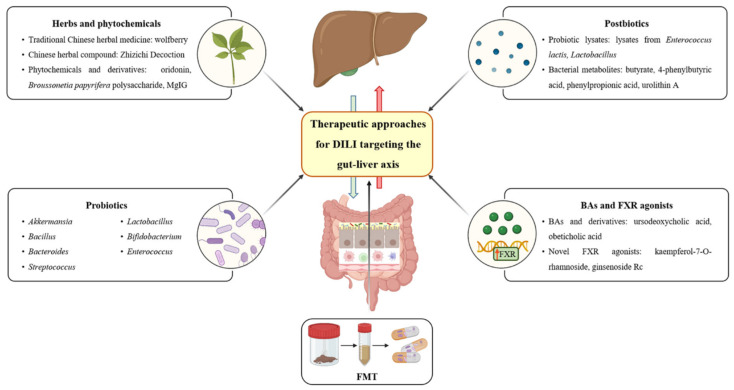
Therapeutic approaches for DILI targeting the gut–liver axis. BA, bile acid; DILI, drug-induced liver injury; FMT, fecal microbial transplantation; FXR, Farnesoid X receptor (Created using BioRender.com (accessed on 28 November 2023)).

DILI is difficult to diagnose due to the lack of special biomarkers, and the most important first action for patients with suspected DILI is to stop taking the implicated drug [13]. An accumulating number of studies have demonstrated that the bidirectional communication between the gut and the liver is involved in the onset and development of DILI. These studies explored the elements of the gut–liver axis altered in DILI, providing the possibilities for interventions in DILI. Herbs and phytochemicals, probiotics, fecal microbial transplantation (FMT), postbiotics, Bas, and FXR agonists target the gut–liver axis, and are becoming novel therapeutic approaches for DILI (Figure 2).

### 3.1. Herbs and Phytochemicals

In recent years, the development and application of herbs and phytochemicals has become a research hotspot. Multiple studies have shown that the hepatoprotective activities of various herbs and phytochemicals were closely related to their regulatory effects on the gut–liver axis [70,71]. 

Wolfberry (*Lycium barbarum* L.) is a traditional Chinese medicine, and it is also widely used as a food supplement. Liu et al. demonstrated that wolfberry could promote the proliferation of *Akkermansia muciniphila* in vitro [72]. It has also been found that wolfberry promoted the recovery of liver injury induced by APAP in mice by enriching the abundance of *Akkermansia muciniphila* in the colon and upregulating Yes-associated protein 1 expression in the liver. Another study found that the hepatotoxicity of Zhizichi Decoction, which is composed of *Gardeniae Fructus* and *Semen Sojae Praeparatum*, was lower than *Gardeniae Fructus* alone, and the mechanism included the improvement of gut microbiota dysbiosis and the restoration of caecal butyric acid content [47].

Oridonin is a phytochemical derived from *Rabdosia rubescens*, and it could lower APAP-induced hepatotoxicity by increasing the abundance of *Bacteroides vulgatus* and upregulating tight junction expression [73]. Magnesium isoglycyrrhizinate (MgIG) is the magnesium salt synthesized from 18-β glycyrrhizic acid, which is extracted from the Chinese traditional medicine glycyrrhiza. Xia et al. reported that MgIG treatment could reshape the gut microbiota composition by increasing the abundance of *Lactobacillus* and decreasing the abundance of *Muribaculaceae*, thereby improving the intestinal barrier function and inhibiting the bacterial translocation, attenuating DILI induced by methotrexate in mice [43]. Gong et al. found that intraperitoneal injection of MgIG alleviated anti-tuberculosis-drug-induced liver injury by recovering the abundance of *Lactobacillus*, enhancing gut barrier function, and inhibiting the activation of the LPS/TLRs/NF-κB pathway [74]. In addition, Xu et al. reported that *Broussonetia papyrifera* polysaccharide alleviated APAP-induced liver injury, inhibited hepatic apoptosis, inflammation, and oxidative stress, and improved hepatic detoxification toward APAP via decreasing intestinal flora disorder [29]. Moreover, polysaccharides derived from *Angelica sinensis*, brown seaweeds, *Pinus koraiensis* pine nut, *Sagittaria sagittifolia*, *Schisandra chinensis*, and *Hippophae rhamnoides* could also protect against DILI; however, the relationship between their hepatoprotective activities and prebiotics effects was not stated [75,76,77,78,79,80].

Based on the aforementioned findings, herbs and phytochemicals targeting the gut–liver axis are promising therapeutic approaches for DILI (Table 2). It is noteworthy that the biologically active compounds of herbal medicines may alter the pharmacokinetics and/or pharmacodynamics of the drugs, thereby affecting their hepatotoxicity [81]. Therefore, the in-depth mechanisms need to be further explored to promote the clinical application of herbs and phytochemicals.

### 3.2. Probiotics

Probiotics are live microorganisms that can exert beneficial effects on human beings, and are involved in the promotion of the digestion and absorption of nutrient substances, preventing the production of toxic metabolites, restoring the balance of the gut microbiota, and maintaining the integrity of the intestinal barrier [82]. As outlined earlier, gut microbiota dysbiosis participates in the progression of DILI, so restoring the gut microbiota balance with probiotics seems to be a promising approach to treat DILI.

*Akkermansia muciniphila* belongs to the Verrucomicrobia phylum, and it is a strictly anaerobic Gram-negative bacterium, constituting more than 1% of the total gut microflora in human beings [83]. *Akkermansia muciniphila* has been considered as a biomarker for a healthy intestine, because of the relativity between its abundance and several intestinal diseases [83]. Moreover, *Akkermansia muciniphila* can be used as a highly promising probiotic for the prevention and treatment of multiple diseases, including obesity, diabetes mellitus, NAFLD, inflammatory bowel disease, and cancers [84]. Xia et al. found that *Akkermansia muciniphila* ameliorated APAP-induced liver injury in mice, as evidenced by the restoration of increased serum levels of ALT and AST, as well as attenuating inflammatory response and oxidative stress in the liver [28]. Additionally, its hepatoprotective effect was closely associated with altered gut microbiota, enhanced gut barrier function, reduced LPS leakage, and promoted SCFA secretion [28].

Many species of *Lactobacillus* have been applied as probiotics to maintain human health and prevent disease. Animal studies have shown that *Lactobacillus acidophilus LA14*, *Lactobacillus rhamnosus* GG, *Lactobacillus ingluviei* ADK10, and *Lactobacillus vaginalis* could alleviate APAP-induced liver injury [85,86,87,88]. Oral administration of *Lactobacillus* species was effective in the prevention of methotrexate-induced liver injury in mice by repairing intestinal barrier function and inhibiting LPS/TLR4-mediated hepatic inflammation [43]. *Lactobacillus casei* and *Lactobacillus Rhamnosus JYLR-005* exerted protective effects against anti-tuberculosis-drug-induced liver injury by decreasing intestinal permeability and LPS translocation [74,89].

In addition, a mixture of several *Bacillus* species spores decreased the serum levels of AST, ALT, proinflammatory cytokines, and ZO-1 in APAP-treated rats [90]. Several other single strains, including *Bifidobacterium longum* R0175, *Bacteroides vulgatus*, *Enterococcus lactis IITRHR1*, and *Streptococcus salivarius*, are potential probiotics that could prevent APAP-induced liver injury [73,91,92,93]. *Streptococcus salivarius* was also effective in the alleviation of diclofenac-induced liver injury in rats [94].

The summary of the hepatoprotective effect of the aforementioned probiotics against DILI is presented in Table 3.

### 3.3. FMT

FMT is an approach to normalizing the gut microbiota composition and restoring a healthy gut microbial environment by transplanting microbial flora from a healthy donator into the intestinal tract of a sick recipient. Since the successful application of FMT in the treatment of *Clostridium difficile* infection, which is a recurrent and refractory disease, growing evidence has demonstrated the efficacy of FMT in treating other diseases, such as inflammatory bowel disease, various metabolic disorders, and neurological diseases [95,96,97,98]. Of late, FMT has also been considered a promising approach to treating liver diseases [99]. Xu et al. transplanted fecal microbiota from *Broussonetia papyrifera* Polysaccharide + APAP-treated mice into recipient mice who previously received antibiotics, and found that FMT could alleviate APAP-induced liver injury by restoring the balance of the intestinal flora [29]. Xia et al. transplanted fecal microbiota from control or methotrexate or MgIG + methotrexate-treated mice into recipient mice who previously received antibiotics [43]. It has been found that the mice that received fecal microbiota from methotrexate-treated mice had increased serum levels of ALT and AST, elevated hepatic inflammation, and decreased tight junctions and E-cadherin expressions compared to the mice that received fecal microbiota from control mice [43]. However, the mice that received fecal microbiota from MgIG + methotrexate-treated mice had decreased liver injury and intestinal permission than the mice that received fecal microbiota from methotrexate-treated mice [43]. Additionally, Hong et al. found that liver injury in mice that received fecal microbiota from Oridonin + APAP-treated mice was less severe than in mice that received fecal microbiota from APAP-treated mice [73]. These findings imply that FMT could restore gut microbiota homeostasis, enhance intestinal barrier function, and lead to an improvement in hepatic function parameters, thereby alleviating the occurrence and development of DILI.

### 3.4. Postbiotics

Postbiotics are defined as “a preparation of inanimate microorganisms and/or their components that confers a health benefit on the host” by the International Scientific Association for Probiotics and Prebiotics (ISAPP) in 2021 [100]. Although they lack live microorganisms, postbiotics showed comparable or even better beneficial effects on host health than probiotics [101]. Additionally, the longer stability of postbiotics makes them more economically feasible than probiotics [102].

In vitro studies demonstrated that the lysates from probiotics *Enterococcus lactis* IITRHR1 and *Lactobacillus acidophilus* MTCC447 could inhibit APAP-induced hepatotoxicity, as evidenced by the elevated cell viability, reduced levels of oxidative stress-related biomarkers, and decreased apoptotic cell death in primary rat hepatocytes [103]. In addition, the lysates from *Lactobacillus fermentum* BGHV110 could alleviate APAP-induced hepatotoxicity by enhancing PINK1-dependent autophagy in human HepG2 cells [104]. Although the protective roles of inanimate microorganisms against drug-induced hepatotoxicity have been demonstrated in vitro, their beneficial effects in vivo and the underlying mechanism still need to be further studied.

As mentioned above, SCFAs are important metabolic products of the gut microbiota. Evidence from both in vitro and in vivo studies has shown that butyrate supplementation could attenuate hepatocyte injury induced by drugs, including genipin and valproate [47,52]. 4-phenylbutyric acid (4-PBA) is a butyric acid derivative naturally produced by colonic bacteria during the fermentation process and is an endoplasmic reticulum stress inhibitor [105]. It has been demonstrated that both pretreatment and post-treatment with 4-PBA could decrease APAP-induced hepatotoxicity in mice, as evidenced by the reduced levels of serum parameters related to hepatic function, decreased hepatocellular apoptosis, necrosis, and DNA fragmentation, and the underlying mechanism might involve the inhibition of endoplasmic reticulum stress [106,107]. Urano et al. found that pretreatment with 4-PBA could alleviate liver injury in DILI mice exposed to fasiglifam, a candidate drug for type 2 diabetes, as well as inhibiting the fasiglifam-induced decrease in cell viability in HepG2 cells [108]. 4-PBA also could ameliorate hepatotoxicity induced by anti-tuberculosis drugs, including pyrazinamide and rifampicin, both in vivo and in vitro [109,110,111]. Phenylpropionic acid (PPA) is a gut microbial metabolic product of L-phenylalanine. Cho et al. found that Jackson Laboratory (6J) mice and germ-free (GF) mice that received fecal microbiota from 6J (6J_GF_) mice had lower susceptibility to APAP-induced hepatotoxicity and higher levels of PPA in serum and cecal contents than Taconic Biosciences (6N) mice and 6N_GF_ mice, respectively [112]. Further study found that PPA-supplemented 6N mice exhibited lower APAP-induced hepatotoxicity than untreated 6N mice, indicating that PPA is a promising gut bacterial metabolite that alleviates APAP-induced hepatotoxicity [112]. Urolithin A is a gut microbial metabolic product of ellagitannins, and it could alleviate APAP-induced hepatic oxidative stress and necrosis in mice, and inhibit APAP-induced cytotoxicity in normal human hepatic cell line L02 [113].

Postbiotics have been reported to favor the improvement of the intestinal epithelial barrier and gut microbial composition. The abovementioned postbiotics, including inanimate microorganisms and bacterial metabolites, have hepatoprotective effects against DILI; however, it is still unclear whether these effects are related to their regulatory effects on the gut–liver axis, and further studies are needed.

### 3.5. BA and FXR Agonists

As mentioned above, impaired BA metabolism and transport might partially contribute to the progression of DILI, so restoring BA homeostasis seems to be an attractive approach to the treatment of DILI.

Ursodeoxycholic acid (UDCA) is a naturally occurring hydrophilic BA widely used in the treatment of PSC and PBC [19]. Recently, Robles-Díaz et al. systematically reviewed the related clinical studies and case reports and found that in 6 out of 8 clinical studies and 18 out of 25 case reports, UDCA was reported to be effective in the prevention or treatment of DILI. Similarly, preclinical experimental results showed that UDCA had hepatoprotective effects against DILI induced by anti-tuberculosis drugs and ceftriaxone [114,115].

Obeticholic acid (OCA) is a derivative of primary BA CDCA and an agonist of FXR [116]. Gai et al. found that OCA could ameliorate hepatic lipid accumulation and oxidative stress induced by valproic acid in both mice and human hepatoma cell line Huh-7 [117]. In addition, OCA has been reported to alleviate liver injury in DILI animals exposed to pyrazinamide or *Tripterygium wilfordii* preparations (drugs for rheumatoid arthritis) by improving BA metabolism disorder [118,119]. Moreover, OCA was effective in the prevention of hepatic dysfunction and inflammation by improving BA homeostasis and normalizing gut microbiota composition in DILI mice induced by methamphetamine (an addictive psychostimulant) [120].

Recently, several novel FXR agonists have been demonstrated to be effective in the treatment of DILI. Liu et al. found that kaempferol-7-O-rhamnoside could bind to FXR and upregulate FXR gene expression to increase cell viability, enhance liver function, and ameliorate oxidative stress in APAP-treated human L02 hepatocytes [121]. Zhong et al. reported that ginsenoside Rc could alleviate APAP-induced hepatotoxicity, inflammation, oxidative stress, and apoptosis by upregulating FXR expression in mice and mouse primary hepatocytes [122].

Based on the aforementioned findings, BA and FXR agonists targeting BA metabolism are attractive therapeutic approaches for DILI, whereas their effect on elements of the gut–liver axis other than BAs, as well as the underlying mechanism, need to be further studied.

## 4. Conclusions and Future Perspectives

There are thousands of drugs that can induce direct, idiosyncratic, or indirect liver injury; however, the involved mechanism is complex and still not completely clear. An accumulating number of studies have demonstrated that DILI patients and animals have altered compositions of the gut microbiota, increased intestinal permeability and LPS translocation, decreased SCFA production, and disrupted BA metabolism homeostasis. In turn, gut–liver axis dysfunction has an influence on the hepatotoxicity of related drugs and the progression of DILI. The diagnosis of DILI is difficult, and the number of therapeutic approaches is limited. Notably, due to the advances in knowledge of the bidirectional communication between the gut and the liver, the gut–liver axis has become a novel therapeutic target for DILI. The therapeutic approaches based on the gut–liver axis, including herbs and phytochemicals, probiotics, FMT, postbiotics, and BA and FXR agonists, have a great potential to ameliorate the severity of DILI. However, the corresponding clinical studies are scarce, and the safety and duration of these treatments still need further studies.

## Figures and Tables

**Table 1:** The alterations in gut microbiota in DILI.

Groups	Drugs	Samples	DILI-Enriched Taxa	DILI-Decreased Taxa	Authors
DILI patients vs. healthy controls	Herbs or/and conventional drugs	Feces	Phylum: Proteobacteria, Actinobacteria	Phylum: Firmicutes Class:Clostridia Order: Clostridiales Genus: *Bacteroides*, *Bifidobacterium*	Zhao et al. (2022) [25]
DILI patients vs. healthy controls	Dietary supplements, conventional drugs	Feces	Phylum: Bacteroidetes	Phylum: Firmicutes Genus: *Acetobacteroides*, *Blautia*, *Caloramator*, *Coprococcus*, *Flavobacterium*, *Lachnospira*, *Natronincola*, *Oscillospira*, *Pseudobutyrivibrio*, *Shuttleworthia*, *Themicanus*, *Turicibacter*	Rodriguez-Diaz et al. (2022) [26]
Treat Graves’ Disease patients vs. initial Graves’ Disease patients	The antithyroid drugs	Feces	Genus: *Eubacterium_rectale*, *Romboutsia*, *Dorea*	Genus: *Faecalibacterium*, *Clostridium_sensu_stricto_1*	Sun et al. (2020) [27]
Sprague Dawley rats, DILI vs. control	The antithyroid drugs	Feces	Phylum: Bacteroidetes, Proteobacteria, and Spirochaetae Genus: *Clostridium_sensu_stricto_1*, *Prevotellaceae_UCG-003*, *Oscillibacter*	Phylum: Firmicutes Genus: *Lactobacillus*, *Romboutsia*, *Faecalibacterium*	Sun et al. (2020) [27]
C57BL/6 mice, DILI vs. control	APAP	Feces	Phylum: Deferribacteres, Cyanobacteria, Desulfobacterota Genus: *Bacteroides*, *Oscillibacter*, *Mucispirillum*, *Colidextribacter*	Phylum: Actinobacteria Genus: *Dubosiella*, *Lactobacillus*, *Bifidobacterium*, *Prevotellaceae_UCG-001*, *Candidatus_Saccharimonas*	Xia et al. (2022) [28]
Kunming mice, DILI vs. control	APAP	cecum contents	Phylum: Deferribacterota Genus: *Enterococcus*, *Bacteroides*, *norank_f_norank_o_ Clostridia_UCG-014*, *Erysipelatoclostridium*, *Blautia*, *Colidextribacter*, *Gordonibacter*, *Eubacterium_fissicatena_group*, *norank_f_Eubacterium_coprostanoligenes_group*, *Eubacterium _nodatum_group*, *Family_XIII_AD3011_group*, *Eubacterium_brachy_ group*, *Oscillibacter*	Phylum: Firmicutes Genus: *Lactobacillus*, *Odoribacter*	Xu et al. (2022) [29]
Kunming mice, DILI vs. control	Methotrexate	Colonic contents	Phylum: Deferribacterota Genus: *Staphylococcus*, *Enterococcus*, *Collinsella*, *Streptococcus*, *Aerococcus*	Phylum: Bacteroidota, unclassified_k_norank_d_Bacteria, Fusobacteriota Genus: *Lactobacillus*, *Ruminococcus*, *norank_f_Muribaculaceae*, *unclassified_f_Lachnospiraceae*, *norank_f_Lachnospiraceae*, *A2*, *Eubacterium_xylanophilum_group*, *Phascolarctobacterium*, *Bifidobacterium*, *Faecalibaculum*	Wang et al. (2022) [31]
Lister hooded rats, strong responders vs. non-responders	Tacrine	Feces	Genus: *Bacteroides*, *Enterobacteriaceae*	Genus: *Lactobacillus*	Yip et al. (2018) [32]
C57BL/6 mice, DILI vs. control	Triclosan	Feces	Phylum: Proteobacteria Family: Enterobacteriaceae	Phylum: Firmicutes, Bacteroidetes Genus: *Bacteroides*, *Blautia*, *Eubacterium*, *Clostridium*, *Roseburia*	Zhang et al. (2022) [33]

APAP, acetaminophen; DILI, drug-induced liver injury; vs. versus.

**Table 2:** Effect of herbs and phytochemicals on DILI by modulation of the gut–liver axis.

Therapeutic Intervention	Research Subjects	Major Findings Related to Liver Injury	Changes in Gut–Liver Axis	Authors
Wolfberry	APAP-treated mice	Decreasing hepatic ALT and AST activities, inhibiting hepatic pathological injury and inflammation	Increasing *Akkermansia muciniphila*, decreasing hepatic LPS content	Liu et al. (2023) [72]
Zhizichi Decoction	*Gardeniae-Fructus*-treated rats	Reducing weight loss, decreasing serum ALT, AST, and total bilirubin, inhibiting hepatic pathological injury	Increasing *Lactobacillus*, *Romboutsia*, *Akkermansia*, and *Prevotella*, decreasing *Enterococcus* and *Parasutterella*, restoring caecal butyric acid content	Luo et al. (2021) [47]
Oridonin	APAP-treated mice	Decreasing serum ALT and AST, inhibiting hepatic centrilobular necrosis, inflammation, and oxidative stress, attenuating the hepatic urea cycle dysregulation, activating Nrf2 pathway	Increasing *Bacteroides vulgatus*, upregulating ZO-1 and occludin expressions	Hong et al. (2021) [73]
MgIG	Methotrexate-treated Mice	Reducing weight loss and liver index, decreasing serum ALT and AST, inhibiting hepatic pathological injury and inflammation	Increasing *Lactobacillus*, decreasing *Muribaculaceae*, improving colonic pathological injury and inflammation, decreasing FITC-dextran leakage, upregulating ZO-1, claudin-1, and E-cadherin expressions, preventing bacterial migrating to the liver	Xia et al. (2022) [43]
MgIG	anti-tuberculosis-drug-treated mice	Decreasing serum ALT, AST, and ALP, inhibiting hepatic pathological injury, inflammation, and oxidative stress, inhibiting TLRs/NF-κB pathway	Increasing *Lactobacillus*, upregulating ZO-1 and occludin expressions, reducing colonic pathological injury, decreasing serum LPS and FITC-dextran	Gong et al. (2022) [74]
*Broussonetia papyrifera* polysaccharide	APAP-treated mice	Decreasing serum ALT and AST, inhibiting hepatic pathological injury, inflammation, and oxidative stress, necrosis, and apoptosis, activating Nrf2 pathway, improved hepatic detoxification ability to APAP	Increasing *Alloprevotella*, *Corynebacterium*, *Jeotgalicoccus*, *Paenochrobactrum* and *Prevotellaceae_UCG-001*, decreasing *Candidatus_Stoquefichus*, *Enterorhabdus*, *Erysipelatoclostridium*, *Eubacterium_brachy_group*, *Eubacterium_nodatum_group*, *Family_XIII_AD3011_group*, *Gordonibacter, norank_f_Eggerthellaceae*, *norank_f_Eubacterium_coprostanoligenes_group* and *norank_f_norank_o_Clostridia_UCG-014*	Xu et al. (2022) [29]

ALP, alkaline phosphatase; ALT, alanine aminotransferase; APAP, acetaminophen; AST, aspartate aminotransferase; FITC, fluorescein isothiocyanate; LPS, lipopolysaccharide; MgIG, magnesium isoglycyrrhizinate; NF-κB, nuclear factor kappa B; Nrf2, nuclear factor E2-related factor 2; TLR4, Toll-like receptor 4; ZO-1, Zonula occludens 1.

**Table 3:** Summary of the hepatoprotective effect of probiotics against DILI.

Therapeutic Intervention	Research Subjects	Major Findings Related to Liver Injury	Changes in Gut–Liver Axis	Authors
*Akkermansia muciniphila*	APAP-treated mice	Decreasing serum ALT and AST, reducing hepatocyte necrosis, inhibiting hepatic inflammation, oxidative stress, and apoptosis, activating PI3K/Akt pathway	Increasing *Lactobacillus*, *Candidatus_Saccharimonas*, and *Akkermansia*, decreasing *Oscillibacter*, upregulating occludin, claudin, and MUC2 expressions, reducing serum LPS, increasing fecal SCFAs concentrations	Xia et al. (2022) [28]
*Lactobacillus acidophilus LA14*	APAP-treated mice	Increasing serum total protein, decreasing serum AST, cholinesterase, and total bilirubin, reducing hepatic pathological injury	Decreasing serum total BAs	Lv et al. (2021) [85]
*Lactobacillus rhamnosus* GG	APAP-treated mice	Decreasing serum ALT, inhibiting hepatic pathological injury, necrosis, and oxidative stress, activating Nrf2 pathway	Decreasing serum FITC-dextran, upregulating ZO-1 expression	Saeedi et al. (2020) [86]
*Lactobacillus ingluviei* ADK10	APAP-treated rats	Reducing oxidative stress in liver and serum	-	Mandal et al. (2013) [87]
*Lactobacillus vaginalis*	APAP-treated mice	Decreasing plasma ALT and AST, reducing systemic inflammation, inhibiting hepatic pathological injury, inflammation, and cell death	-	Zeng et al. (2023) [88]
*Lactobacillus* species	Methotrexate-treated mice	Reducing hepatic pathological injury, inhibiting inflammation in liver and serum	Reducing colonic pathological injury, FITC-dextran leakage	Xia et al. (2022) [43]
*Lactobacillus casei*	anti-tuberculosis-drug-treated mice	Decreasing serum ALP, recovering hepatic lobule, reducing hepatocyte necrosis, alleviating hepatic inflammation and oxidative stress, inhibiting TLR4/NF-κB/MyD88 pathway	Increasing *Lactobacillus* and *Desulfovibrio*, decreasing *Bilophila*, reducing serum LPS, upregulating ZO-1 and claudin-1 expressions	Li et al. (2023) [89]
*Lactobacillus Rhamnosus JYLR-005*	anti-tuberculosis-drug-treated mice	Decreasing serum ALT and AST, inhibiting hepatic pathological injury, inflammation and oxidative stress, inhibiting TLRs/NF-κB pathway	Decreasing serum LPS and FITC-dextran	Gong et al. (2022) [74]
*Bacillus* species spores	APAP-treated rats	Decreasing serum ALT and AST, reducing systemic inflammation and oxidative stress, inhibiting hepatic pathological injury	Reducing serum ZO-1	Neag et al. (2020) [90]
*Bifidobacterium longum* R0175	APAP-treated mice	Decreasing serum ALT and AST, inhibiting hepatic pathological injury, inflammation, hepatocyte death, and oxidative stress, activating Nrf2 pathway	Increasing *Firmicutes*, *Lactobacillaceae*, *Lactobacillus* and *Blautia*, decreasing *Rikenellaceae*, *Rikenellaceae RC9*, *Lachnospiraceae NK4A136*, and *Alistipes*, altering microbiota-derived metabolites, increasing metabolite sedanolide	Li et al. (2023) [91]
*Bacteroides vulgatus*	APAP-treated mice	Decreasing serum ALT and AST, inhibiting hepatic centrilobular necrosis, inflammation, and oxidative stress, attenuating the hepatic urea cycle dysregulation, activating Nrf2 pathway	-	Hong et al. (2021) [73]
*Enterococcus lactis IITRHR1*	APAP-treated rats	Decreasing serum ALT, AST, and ALP, inhibiting hepatic pathological injury, hepatic apoptosis, oxidative stress, and DNA damage	-	Sharma et al. (2012) [92]
*Streptococcus salivarius*	APAP-treated rats	Decreasing serum ALT, AST, and ALP, inhibiting hepatic oxidative stress	-	Riane et al. (2019) [93]
*Streptococcus salivarius*	Diclofenac-treated rats	Decreasing serum ALT, AST, and ALP, inhibiting hepatic pathological injury, hepatic oxidative stress	-	Riane et al. (2020) [94]

ALP, alkaline phosphatase; ALT, alanine aminotransferase; APAP, acetaminophen; AST, aspartate aminotransferase; BA, bile acid; FITC, fluorescein isothiocyanate; LPS, lipopolysaccharide; MUC2, mucin-2; MyD88, myeloid differentiation factor 88; NF-κB, nuclear factor kappa B; Nrf2, nuclear factor E2-related factor 2; SCFAs, short-chain fatty acids; TLR4, Toll-like receptor 4; ZO-1, Zonula occludens 1.
